# Observations on the Lymphatic Distension of the Lower Extremities of Women, While in the Puerperal State

**Published:** 1810-04

**Authors:** 


### *Observations on the Lymphatic Distension of the Lower Extremities of Women, while in the Puerperal State.* By Dr. Wyer.

[ Communicated to the Massachusetts Medical Society. ]

WRITERS on this disease have imputed it to a variety of causes, some to taking cold, others to a rheumatic affection, and others have had recourse to a dropsical disposition, to help them out in their attempts to account for it;

Mauriceau

Mauriceau has charged it to a reflux of the lochia upon the part, and not a few authors have deemed it to be a metastasis lactea. In the cases I have met with, there has been no reason to suspect either of the above causes, the lochial discharge and lacteal secretions particularly, having in all been so perfectly in their natural state, both at the attack, and under the complaint, that I could not think there was a shadow of reason to support an opinion, that either of these had the smallest influence in producing the disease. When I inform you I have had five cases of this disorder under my care, one of them an amiable wife, whose sufferings were both lengthy and severe; and that to the whole of them I was very attentive, though I fear but of little use; and if at the same time you will please to recollect in how unsatisfactory a manner it has been hitherto described, how few have accounted for it rationally, or pointed out such methods of relief, as the anxiety and distress it generally occasions, ever lead the Faculty to wish to bestow; and that hitherto, speedy relief has seldom been obtained; these considerations, I doubt not, will so far interest your well known candour in my behalf, as, at least, to secure me from censure in having presumed to touch upon a disorder confessedly involved in obscurity, and on which, I fear, I have not been able to throw much additional light; but as the profession may be benefited in some degree by knowing what has been found useless, as well as what is useful, in particular diseases, I thought the following remarks might not be wholly without their use.

There is much reason to suppose the sex must have been occasionally subject to this disease in all ages, and in every climate; but I have concluded that it is not a very frequent complaint, from having met with only five cases in nine hundred and eighty-nine women I delivered while in business, in which was included every variety of circumstance, which I can any way conceive connected with the obstetric art. The first four cases happened at different seasons of the year, and to women whose circumstances agreed but in few particulars; they had all borne children before, one two, two of them three, and the fourth four children; one was 24, another 26, a third 27, and the fourth 36 years of age; in this lying in, which was followed with the disorder I mean to describe, the labour had been natural, and tolerable easy; in all of them the placenta was delivered within half an hour after the child; neither of them lost much blood, had bad after pains, or

 other other unpleasant symptoms. The disorder made its appearance between the second and third week after delivery, at a time when they thought themselves nearly well enough to go abroad, and at a time when they had not been exposed to any error or accident whatever. They were women who had enjoyed good health previous to delivery, and were free from any constitutional complaint; they all suckled their children, had plenty of milk, and their children were very promising. Two of them complained on the fifteenth, one on the sixteenth, and the other on the nineteenth day, after getting to bed. Stiffness and pain in the upper part of the thigh, accompanied with a sense of lassitude and heaviness, pain in the back, groin, and labium pudendi, and thence downwards through the thigh, leg and foot, were the first symptoms; swelling soon followed in each of them; and in the course of two or three days the whole left limb was greatly enlarged, hard, exquisitely tender, and immoveable; a considerable degree of fever, with thirst, restlesness, and loss of appetite, attended. The pain abated in some degree in the course of the first week, but the swelling and lameness continued for near three months. The pain and swelling abated first in the upper part of the thigh where it began, and finally went off in nearly the same direction in which it had come on. They all finally recovered, and have never since had the least return of their disorder, though three of them have had children since. In these cases, which were very tedious to all concerned, I tried all the various topical applications, (blisters excepted) which I then thought could have the least tendency to relieve. Internally I used nitre, antimonials, mercurials, and bark, as symptoms appeared to require; but from the most careful attention to the effects produced by both the internal and external remedies, I am not able to say that I think any thing I did was of much service. Opiates for a time would abate the pain and procure rest; the faltering state of the powers of nature induced me to use the bark with all the patients, and I really thought the irregular remitting fever, which attended the oldest, was abated, her strength supported, and appetite mended by this medicine; but the disease in the limb was proof against both bark and opium. When they began to recover, I was as much at a loss for a cause of the change, as when they were taken down; and I believe the discovery of the one will be the best guide to the other.

In my fifth case, in which I felt myself tenderly interested terested both as physician and friend, the labour was in the middle of a very cold winter; it was of the natural kind, but very severe; the delivery of the placenta was attended with much pain and great flooding, the after pains were very violent, and were followed at the end of the second day with the most painful hæmorrhoids I had ever known. These however, by anodyne and emollient applications, were but of short continuance; from their abatement to the end of the eighth day, nothing further occurred worth mentioning; at this time, my patient appeared in a fair way to do well, her appetite was good, bowels regular, she rested well, had plenty of milk, and was daily gaining strength; appearances however, soon began to change; she first complained towards night on the tenth day from delivery, of a sudden sense of weariness, while sitting in an easy chair, accompanied with a sensation in perinæo extending to the middle of the glutæi muscles, like what might have been expected from sitting upon a hard unyielding seat; her pulse soon quickened, and she became very thirsty, with a bad taste in the mouth; she lay down expecting relief from changing her posture; but in this she was disappointed; the pains increased very rapidly, and every posture was alike distressing. In eight hours from the attack, the pain had extended up the inside of the pubis, and thence to the inguinal glands, which soon became greatly tumefied, and so very painful and tender, as to be incapable of bearing the lightest pressure of the bed-clothes; from these glands the pain in twenty-four hours extended down through the thigh in the course of the principal lymphatics to the knee; from the knee, it still was confined to the lymphatics, and through them passed so rapidly to the toes, as to have disabled the whole limb in thirty-six hours. In this stage of the disorder, which I think might without great impropriety be denominated the inflammatory, the pulse was seldom below a hundred and twenty, and very hard; the whole surface of the body was hot and dry; the heat of the limb was particularly increased, but the colour was as yet unchanged; frequent discharges from the bowels now took place, and a few hours after a constant vomiting of dark coloured bile. By the use of acescent drinks and an anti-emetic mixture of succus limonum, sal absynthii, and aquæ menthæ, the stomach and bowels were quieted; and by plentiful dilution afterwards, accompanied with nitre and antimonials, the skin was opened, and a plentiful perspiration following, the heat and fever abated.

 I again

I again examined the limb very carefully, and found neither change of colour nor increased size, but from the hip to the toes such tenderness and stiffness as rendered it wholly useless; in two days more the pain began to abate, but in its place an enlargement at the upper part of the thigh was perceivable; the swelling extended downwards by degrees till the third day from its beginning, at which time the whole thigh, leg and foot were distended in every part to three times their natural size; the limb had a tense smooth feel, did not retain the impression of the finger, was a little hotter than the other, but had the most remarkable glossy whiteness I had ever before seen in any subject. On the side of the tibialis anticus muscle, I could discover a slight blush of red, as if a straw dipped in raspberry juice had been drawn from the knee to the top of the foot; this stripe was very painful to the touch, particularly just below the middle of the muscle, where is seated a large lymphatic gland. I endeavoured to contrive an easy posture for the limb, but in this, and my expectations that keeping it parallel to the horizon would diminish the swelling, I was equally disappointed. From the size and stiffness of the limb, and weakness of the whole body induced by the preceding fever, pain, and evacuations, she was unable to sit up, or to afford herself the least assistance in bed. The vomiting returned again; and from the taste in the mouth, the colour and quantity of what was brought up, I was convinced the biliary secretion was greatly increased; the pulse again quickened, but had lost its hardness; the skin was soft and moist; her spirits were much depressed, and every power of Nature seemed to faulter; no kind of nourishment could be taken, for every thing offered, either as food or medicine, excited nausea, such things excepted as were of the ascescent kind; oranges, lemons, and cyder, alone were grateful, and I allowed them freely; at the end of two days from the last attack of vomiting, the stomach became quiet, and could bear a little sago with orange juice, and her mind was somewhat more reconciled to her fatiguing lameness; the limb still, however, retained its size and immobility.

Matters continued in this state for thirty-four days with no change of sufficient importance to deserve attention. I now found the swelling beginning to subside at the upper part of the thigh, in the inguinal glands, and by degrees downwards to the foot. That its decline was very slow may be concluded from its having scarcely fallen to its its natural size in three months; when the swelling was pretty well reduced, excepting in the foot, which continued much as it had been. At last it began to yield, and in ninety-seven days from the attack, motion was so far restored to the knee and ancle as to enable her to raise the limb and to place it in different postures, without assistance, but as yet it could not support the weight of the body; by degrees strength was so far restored as to enable her to hobble about the chamber, and in twelve days more she could walk in the street. Being desirous of enjoying a share of the exercise she long wanted, she was induced to walk about a quarter of a mile; upon the approach of evening she perceived the ancle beginning to swell again, the tenderness and red stripe upon the anterior surface of the leg began to appear, and great were her apprehensions of a return of the disease; but by the immediate application of a blister to the most inflamed part, it got better in a few days; and excepting some little uneasiness upon using much exercise, with a little swelling upon keeping the limb long in one posture, it appeared to be restored to its natural and healthy state.

I shall now give in one point of view, such particulars of my treatment of this tedious disorder, as appear most worthy of notice.

In the first stage of the disease the very great quantities of bile thrown upon the first passages, occasioning violent vomiting and purging, evidently required the first attention. Frequent draughts of chamomile tea to wash the stomach were first administered; and afterward an anti-emetic mixture of succus limonum, sal absinthii, L. L. and aquæ menthæ abated these symptoms. Antimonial and nitrous medicines with dilution brought on perspiration, and abated the heat and great inquietude which had occurred in the first stage of the complaint. Emolient fomentations, gentle frictions, discutient liniments, saturnine washes, lubricating ointments, were all successively and frequently used to the limb during the continuance of the swelling, but I am ready to acknowledge, that neither of them appeared to be of the least service; the disease appeared determined, [as the nurse expressed it] to have its course, in opposition to the many exertions I daily made to give relief. The violence of the pain at times demanded anodynes; by their use, I was always able to give a night's rest, without any bad consequences on the following day. As cider agreed better with the stomach than any drink I proposed, I allowed it freely; it never gave the the least uneasiness, and very evidently prevented the trouble which was constantly threatened from very large quantities of bile thrown upon the stomach. Her diet consisted of sago, gruel, and ocasionally a little light meat, with broths, &c. Clysters were frequently injected, for the double purpose of keeping the bowels open, and to serve as a fomentation for an enlargement, hardness, and tenderness felt in the vagina, which I doubt not was an over-distended lymphatic gland. As the swelling of the limb subsided, I caused cloths wet with a cold solution of sacch. saturni to be frequently applied, with a view to restore the tone of the lately overdistended fibres; and as often as the stomach would bear it, I gave the bark with elix. vitriol. allowing her to drink freely of warter highly impregnated with fixed air. Increasing appetite, less bile, and more strength, were very evidently the effects of this plan.

For the swelling and return of inflammation in the lymphatic of the leg which were brought on by walking abroad, I applied a blister upon the most inflamed part, being now convinced of the want of efficacy of all remedies hitherto employed. The relief was so sudden and lasting, that I was much inclined to think that had blisters been applied earlier in the disease to different parts of the limb, much suffering would have been prevented, and the cure much sooner completed; the evidence in favour of this opinion appeared to me so strong, that I made up my mind to try it, should I ever again be consulted in a similar case; and I would beg leave to submit it to your judgment, whether it might not be advisable to give this aplication a fair trial, in a disease, acknowledged by all who have seen it, both tedious and vexatious, and which has not as yet yielded, in a short time, to any mode of treatment which has been adopted, and for which, it is generally thought, we are not yet in the possession of an adequate remedy. Perhaps the *blister* may in future be found to be the desideratum we have long wanted.

Many reasons might be adduced in favour of this practice, but as my sole motive in writing this paper, was to lay before the Society a few plan facts, I shall leave reasoning upon them, to those who are better qualified to discuss this subject.

But that this disease depends upon an accumulation of lymph in the limb, and that this accumulation is dependent on causes peculiarly connected with the puerperal state, I think may be proved by the most incontestable arguments, arguments, and will, I doubt not, readily occur to every member of this Society, who will give himself the trouble to reflect on the symptoms, the rise, progress, and termination of this very tedious disease.

I can but lament that it has been so little in my power to throw more light on the curative indications and the best method of answering them, than I have done.

If any thing I have said, should be found to have placed the disease in the least useful point of view, to excite the attention of more judicious practitioners to endeavour to investigate its cause, nature and cure, or to support the spirits of those who are either subjects or spectators of it, it will afford me great pleasure. Some consolation arises from the considerations that this disease has seldom been known to prove fatal; and that although as yet, it cannot be immediately relieved by art, nature generally struggles through the trying conflict, and has so far as either my reading or observation has extended, generally come off completely victorious.

